# Pulmonary transit time is a predictor of outcomes in heart failure: a cardiovascular magnetic resonance first-pass perfusion study

**DOI:** 10.1186/s12872-024-04003-w

**Published:** 2024-06-28

**Authors:** Jonathan Farley, Louise AE. Brown, Pankaj Garg, Ali Wahab, Joel RL. Klassen, Nicholas Jex, Sharmaine Thirunavukarasu, Amrit Chowdhary, Noor Sharrack, Miroslawa Gorecka, Hui Xue, Nigel Artis, Eylem Levelt, Erica Dall’Armellina, Peter Kellman, John P. Greenwood, Sven Plein, Peter P. Swoboda

**Affiliations:** 1 Multidisciplinary Cardiovascular Research Centre, Department of Biomedical Imaging Science, Leeds Institute of Cardiovascular and Metabolic Medicine, University of Leeds, Leeds, LS2 9JT UK; 2 Norwich Medical School, University of East Anglia, Norfolk, UK; 3 National Institutes for Health, National Heart, Lung, and Blood Institute, Bethesda, USA; 4 Department of Cardiology, Mid Yorkshire Hospitals NHS Trust, Wakefield, UK

**Keywords:** Pulmonary transit time, Perfusion imaging, Machine learning, Heart failure

## Abstract

**Background:**

Pulmonary transit time (PTT) can be measured automatically from arterial input function (AIF) images of dual sequence first-pass perfusion imaging. PTT has been validated against invasive cardiac catheterisation correlating with both cardiac output and left ventricular filling pressure (both important prognostic markers in heart failure). We hypothesized that prolonged PTT is associated with clinical outcomes in patients with heart failure.

**Methods:**

We recruited outpatients with a recent diagnosis of non-ischaemic heart failure with left ventricular ejection fraction (LVEF) < 50% on referral echocardiogram. Patients were followed up by a review of medical records for major adverse cardiovascular events (MACE) defined as all-cause mortality, heart failure hospitalization, ventricular arrhythmia, stroke or myocardial infarction. PTT was measured automatically from low-resolution AIF dynamic series of both the LV and RV during rest perfusion imaging, and the PTT was measured as the time (in seconds) between the centroid of the left (LV) and right ventricle (RV) indicator dilution curves.

**Results:**

Patients (*N* = 294) were followed-up for median 2.0 years during which 37 patients (12.6%) had at least one MACE event. On univariate Cox regression analysis there was a significant association between PTT and MACE (Hazard ratio (HR) 1.16, 95% confidence interval (CI) 1.08–1.25, *P* = 0.0001). There was also significant association between PTT and heart failure hospitalisation (HR 1.15, 95% CI 1.02–1.29, *P* = 0.02) and moderate correlation between PTT and N-terminal pro B-type natriuretic peptide (NT-proBNP, *r* = 0.51, *P* < 0.001). PTT remained predictive of MACE after adjustment for clinical and imaging factors but was no longer significant once adjusted for NT-proBNP.

**Conclusions:**

PTT measured automatically during CMR perfusion imaging in patients with recent onset non-ischaemic heart failure is predictive of MACE and in particular heart failure hospitalisation. PTT derived in this way may be a non-invasive marker of haemodynamic congestion in heart failure and future studies are required to establish if prolonged PTT identifies those who may warrant closer follow-up or medicine optimisation to reduce the risk of future adverse events.

## Background

Cardiovascular magnetic resonance (CMR) imaging is often used to investigate the aetiology of heart failure, as well as being used as to inform prognosis. To date, left ventricular ejection fraction has been the main parameter for risk stratification and in guiding decisions on the use of evidence-based medical therapies, as well as device therapies such as implantable cardioverter-defibrillators for the prevention of sudden cardiac death [1]. Multiple CMR parameters have been proposed for prognostication in heart failure, including but not limited to the location and burden of myocardial fibrosis, native T1 time, extracellular volume fraction (ECV), left ventricular end-diastolic volume (LVEDV), left ventricular mass and right ventricular ejection fraction (RVEF) [2, 3].

CMR-derived pulmonary transit time (PTT) and pulmonary blood volume (PBV) have been suggested for prognostication in patients with heart failure [4, 5]. PTT is the time taken for blood to pass through the pulmonary circulation and pulmonary blood volume is the product of PTT and cardiac output. The original non-invasive methodology to measure PTT involved the use of dynamic contrast-enhanced subtracted time-resolved imaging to follow the passage of contrast from the pulmonary artery to the left atrium [6]. In clinical practice, first-pass perfusion imaging is frequently carried out with short-axis imaging of the left and right ventricles, and it is possible to estimate PTT from these images without the requirement for additional imaging or contrast administration [4, 5]. The main limitations of these methodologies are the risk of signal saturation and the necessity for manual segmentation, which can be overcome by using machine learning automated analysis of the low-resolution arterial input function (AIF) from a dual sequence method of quantitative perfusion [7]. Both PTT and PBV have been shown to be markers of global cardiopulmonary function encompassing biventricular function and left ventricular filling pressure, which are important markers in heart failure [8, 9].

Invasively measured pulmonary circulation times have been demonstrated to be associated with heart failure symptoms in the form of New York Heart Association (NYHA) class for over 50 years [10, 11]. However, with advances in non-invasive imaging, invasive assessment of heart failure is now carried out less frequently. With advances in CMR technology and image processing, it is now possible to measure PTT non-invasively and automatically.

We, therefore, aimed to investigate whether prolonged PTT by automated measurement is associated with clinical outcomes in patients with heart failure. Furthermore, we aimed to examine whether this was independent of established prognostic markers in heart failure.

## Methods

### Study Population

Patients were recruited prospectively between 28/2/2018 and 19/2/2020. We recruited outpatients with a diagnosis of heart failure within the last 12 months with LVEF < 50% on referral echocardiogram and aged > 18 years. Patients were typically referred for investigation into the aetiology of heart failure. Prior to the CMR scan, patients were presumed to have non-ischaemic heart failure with patients excluded if they had a history of previous coronary artery disease (stenosis > 70% on invasive coronary angiography), myocardial infarction, coronary revascularisation or symptoms of angina. Other exclusion criteria included hypertrophic cardiomyopathy, amyloidosis, congenital heart disease, suspected acute pathology such as myocarditis, advanced renal failure, or any contraindication to CMR or gadolinium-based contrast agents. For this study we also excluded patients with evidence of prior myocardial infarction on CMR.

Patients underwent a clinical assessment on the day of their CMR appointment, including medical history, New York Heart Association (NYHA) functional class, risk factors and current medications. A blood sample was taken at the same time for assessment of NT-proBNP.

The study protocol was approved by the National Research Ethics Committee (17/YH/0300), and all patients gave written informed consent.

### CMR Protocol

All CMR studies were performed on a Siemens Prisma 3T scanner (Siemens Healthineers, Erlangen, Germany). Participants were advised to avoid caffeine for 24 h before the study. The protocol consisted of cine imaging, stress and rest perfusion imaging (using adenosine stressor), and motion-corrected (MOCO) bright blood late gadolinium enhancement (LGE). The dual sequence single bolus perfusion sequence has been described previously [12]. Perfusion maps were acquired at 3 short axis, 8 mm slices, at the basal, mid and apical levels, with slice spacing varying on a per-patient basis to cover the left ventricle. LGE images were acquired as a short axis stack and in 4, 3 and 2 chamber views. For perfusion imaging, an intravenous bolus of 0.05 mmol/kg gadolinium-based contrast agent (Gadovist, Bayer, Leverkusen, Germany) was administered at 5 ml/s followed by a 20 ml saline flush using an automated injection pump (Medrad MRXperion Injection System, Bayer) for both stress and rest imaging [13]. A top-up of 0.05 mmol/kg gadolinium-based contrast agent was given immediately following rest perfusion imaging.

### CMR Analysis

All CMR studies were analysed using cvi42 software (Circle Cardiovascular Imaging Inc, Calgary, Canada). Endocardial and epicardial borders were drawn, excluding papillary muscles. Left ventricular/ right ventricular volumes, LV mass and left atrium (LA) volumes were indexed to BSA. LGE was reported if enhancement was visualised on two orthogonal planes.

**Fig. 1:**
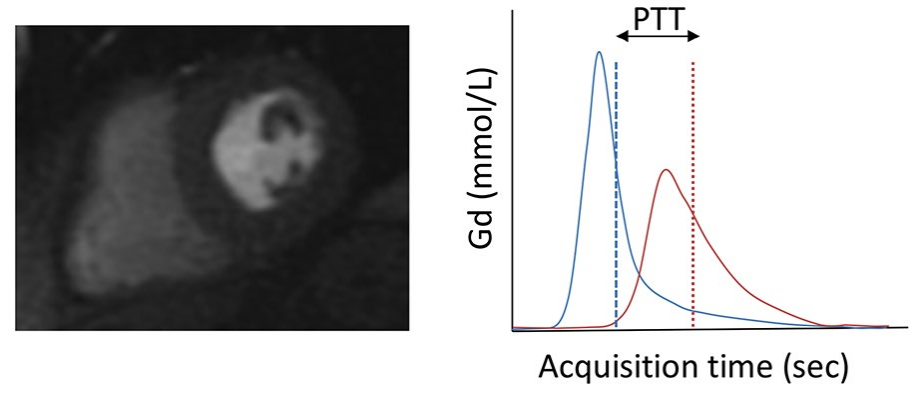
**Panel a |** Rest perfusion image showing contrast in LV blood pool **Panel b |** Calculation of pulmonary transit time (blue curve indicates right ventricle; red curve, left ventricle; blue dash line, RV centroid; red dot line, LV centroid; Gd, Gadolinium concentration)

PTT was measured using Gadgetron from low resolution arterial input function (AIF) rest first-pass perfusion dynamic images of both the LV and RV. A convolutional neural network approach was used to automatically segment both the LV and RV cavities and blood pool signal intensity was measured over time [14]. Gadolinium contrast curves (Fig. 1) were calculated from signal intensity data and the PTT measured as the time (in seconds) between the centroid peaks of the LV and RV curves [15, 16]. The results were automatically fed back into cvi42 software allowing in-line analysis of PTT. Pulmonary blood volume index (PBVi) was calculated as the product of PTT and cardiac output (stroke volume from short axis cine stack x heart rate) indexed to body surface area.

### Follow up

Patients were followed up for a minimum of 12 months by review of electronic clinical records for major adverse cardiovascular events including: cardiovascular death, heart failure admission, non-fatal stroke and non-fatal myocardial infarction. Data on any unplanned hospitalisation and all-cause mortality were both collected but not included in the MACE primary endpoint. Follow up data was available for all patients. Cause of death was obtained by review of death certificates or post-mortem results.

### Statistical analysis

Statistical analysis was performed using SPSS 23 (IBM SPSS, Armonk, NY, USA). Continuous data are presented as median (interquartile range), and categorical data presented as number (percentage). Continuous variables were compared using unpaired (two sample) t-test or Mann-Whitney test depending on the normality of data. Categorical variables were compared using chi-squared test. Correlation was assessed using Pearson correlation. Univariate and multivariate Cox proportional hazards regression was performed with each continuous variable standardized as the z score for the population studied, in order to allow for comparison between variables. Multivariate analysis was performed to assess the association between PTT and MACE after correction for variables that were significant in univariate analysis. A p-value of < 0.05 was considered statistically significant.

## Results

### Study population

A total of 382 patients were prospectively recruited. Following CMR, 17 patients (4.5%) were excluded from this study due to suboptimal LV fit during automated PTT assessment and 71 (18.6%) were excluded due to a new finding of myocardial infarction on LGE imaging, leaving a total of 294 patients with presumed non-ischaemic heart failure.

**Fig. 2:**
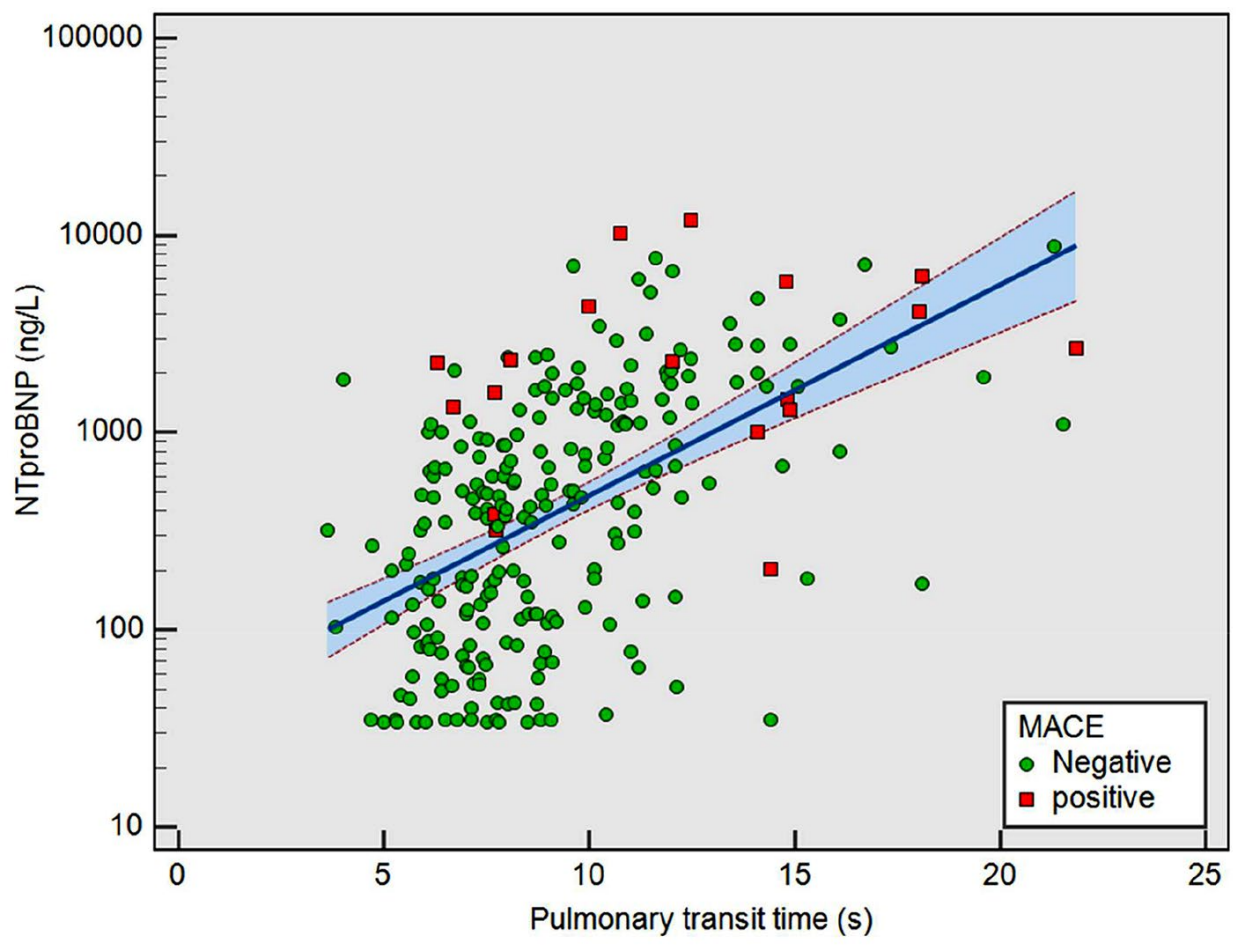
Correlation between Pulmonary Transit Time and log NT-proBNP

In this cohort, median age was 62 years (IQR 54–62), 192 (65%) were male and median left ventricular ejection fraction (LVEF) was 42% (IQR 32–50). 98 patients (33%) had an LVEF < 35%. Median PTT was 8.5s (IQR 7.1–10.8 s). There was a significant association between PTT and log NT-proBNP (*R* = 0.55, *P* < 0.001, Fig. 2). Non-ischaemic LGE was found in 102 (35%) patients and when present affected a median of 2 (IQR 1–3) segments.

### Patient outcomes

Patients were followed-up over a median of 2.0 years (interquartile range 1.3–3.4 years).

During follow-up, 37 patients suffered a major adverse cardiovascular event. Of the individual components, there were 15 heart failure hospitalisations, 4 non-fatal strokes, 3 non-fatal myocardial infarctions and 20 deaths.

Patients who suffered a MACE event, compared to those who did not, were older, had higher NYHA class and increased history of prior TIA/CVA. They also had higher NT-proBNP levels, increased diuretic usage and higher prevalence of peripheral oedema. There was no difference in use of any guideline directed medical therapy (Table 1).

**Table 1:** Clinical characteristics of patients according to MACE

	MACE (*n* = 37)	No MACE (*n* = 257)	*P* value
Age, y	71 (66–77)	60 (52–70)	< 0.0001*
Male sex, n (%)	26 (70)	166 (65)	0.50
BMI, kg/m^2^	27 (25–31)	28 (27–29)	0.61
HR, bpm	75 (66–82)	70 (62–80)	0.08
SBP, mmHg	124 (106–141)	121 (109–135)	0.96
DBP, mmHg	73 (67–83)	74 (69–81)	0.49
NYHA I, n (%)	11 (30)	153 (60)	< 0.0001*
NYHA II, n (%)	15 (41)	81 (32)	
NYHA III, n (%)	11 (30)	20 [8]	
Shortness of breath, n (%)	26 (70)	102 (40)	0.0005*
Orthopnoea, n (%)	6 [16]	42 [16]	0.98
Oedema, n (%)	10 (27)	35 [14]	0.03*
Diabetes, n (%)	8 [17]	37 [14]	0.25
Hypertension, n (%)	16 (43)	115 (45)	0.86
Hypercholesterolemia, n (%)	7 [18]	73 (28)	0.23
Previous TIA/ CVA, n (%)	9 (24)	18 [7]	0.0007*
AF, n (%)	14 (38)	86 (33)	0.61
Current smoker, n (%)	4 [11]	42 [16]	0.38
Previous smoker, n (%)	16 (43)	86 (33)	0.26
Antiplatelet, n (%)	5 [14]	40 [16]	0.27
Betablocker, n (%)	27 (73)	202 (79)	0.24
Statin, n (%)	20 (54)	102 (40)	0.06
ACE-inhibitor/ angiotensin receptor blocker, n (%)	30 (81)	220 (86)	0.25
MRA, n (%)	9 (24)	86 (33)	0.16
Diuretic, n (%)	27 (73)	101 (39)	0.0001*
Anticoagulant, n (%)	15 (41)	84 (33)	0.17
NT-proBNP (ng/L) (*N* = 251)	1595 (495–4285)	432 (119–1137)	< 0.0001*

Values are median (interquartile range) or n (%). *Signifies *p* < 0.05; BMI indicates body mass index; HR, heart rate; SBP, systolic blood pressure; DBP, diastolic blood pressure; NYHA, New York Heart Association Functional Class; TIA, transient ischaemic attack; CVA, cerebrovascular accident AF, atrial fibrillation; MRA, mineralocorticoid receptor blocker

Patients who suffered a MACE event, compared to those who did not had no significant difference in LVEDV, LVEF, right ventricular end diastolic volume (RVEDV), right evntricular ejection fraction (RVEF), LA volume or prevalence of non-ischaemic LGE (Table 2). PTT was significantly longer in those who suffered a MACE event 9.6s (IQR 7.7–14.8 s) vs. 8.4s (IQR 7.0–10.5 s) *P* = 0.008. There was no significant difference in PBVI according to MACE status.

**Table 2:** CMR parameters of patients according to MACE

	MACE (*n* = 37)	No MACE (*n* = 257)	*P* value
LVEDV, ml	190 (153–260)	204 (160–248)	0.79
LVEDVI, ml/m^2^	101 (80–141)	99 (85–123)	0.65
LV mass, g	132 (100–172)	128 (97–157)	0.52
LV mass indexed, g/m^2^	69 (52–91)	67 (54–76)	0.14
LVEF (%)	37 (26–51)	42 (32–50)	0.23
RVEDV, ml	143 (122–172)	147 (121–178)	0.96
RVEDVI, ml/m^2^	75 (66–89)	74 (64–89)	0.86
RVEF (%)	49 (36–57)	50 (42–58)	0.45
LA volume, ml	75 (54–156)	78 (59–111)	0.99
LA volume indexed, ml/ m^2^	41 (30–57)	39 (30–55)	0.57
PTT, s	9.6 (7.7–14.8)	8.4 (7.0-10.5)	0.008*
PBVI, ml/ m^2^	413 (321–568)	398 (324–477)	0.31
Non-ischaemic LGE, n (%)	19 (51)	93 (36)	0.06

Values are median (interquartile range) or n (%). *Signifies *p* < 0.05; LVEDV indicates left ventricular end diastolic volume; LVEDVI, left ventricular end diastolic volume indexed; LVEF, left ventricular ejection fraction; RVEDV, right ventricular end diastolic volume; RVEDVI, right ventricular end diastolic volume indexed; RVEF, right ventricular ejection fraction; LA, left atrial; PBVI, pulmonary blood volume indexed; LGE, late gadolinium enhancement

### Association between PTT and MACE

**Fig. 3:**
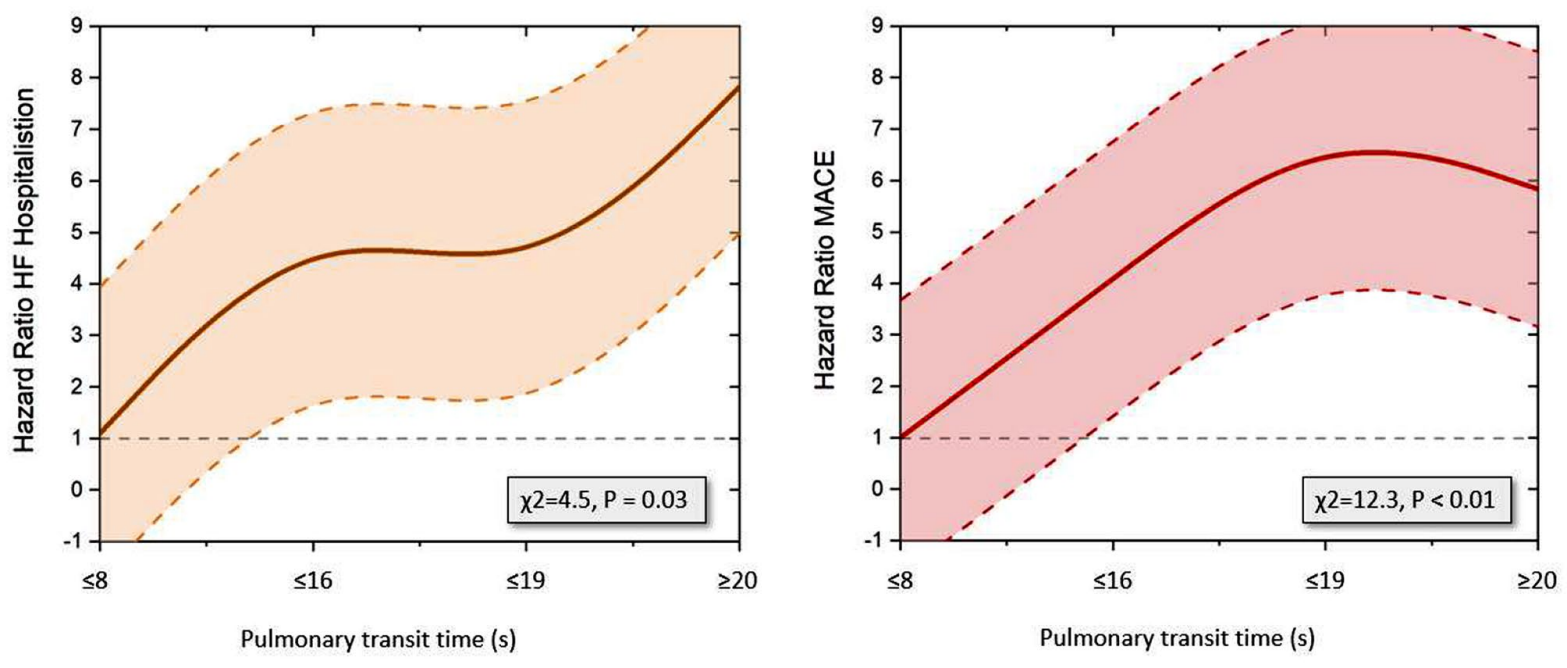
**Left |** Graph showing association between duration of PTT and hazard ratio for heart failure hospitalisation **Right |** Graph showing association between duration of PTT and hazard ratio for MACE

On Cox regression analysis PTT had a significant association with MACE (Hazard ratio (HR) 1.16 (95% confidence interval (CI) 1.08–1.25), *P* = 0.0001, Table 3). PTT also had significant associations with all-cause mortality (HR 1.12, 95% CI 1.00-1.24, *P* = 0.04) and heart failure hospitalisation (HR 1.15, 95% CI 1.02–1.29, *P* = 0.02), Fig. 3.

**Table 3:** Univariate cox-regression analysis of predictors of MACE

Covariate	Beta	SE	HR	95% CI of HR	*P* value
Age	0.081	0.018	1.08	1.05–1.12	< 0.0001*
Male Sex	-0.25	0.36	0.78	0.38–1.58	0.48
BMI	0.0071	0.012	1.01	0.98–1.03	0.54
Heart rate	0.0094	0.011	1.01	0.99–1.03	0.39
Systolic BP	0.0047	0.0092	1.01	0.99–1.02	0.61
Diastolic BP	-0.0079	0.017	0.99	0.96–1.03	0.64
Atrial fibrillation	0.10	0.34	1.11	0.57–2.16	0.76
NYHA class	0.88	0.21	2.41	1.60–3.64	< 0.0001*
Diuretic use	1.34	0.37	3.83	1.85–7.93	0.0003*
NT-proBNP (scaled)	0.38	0.060	1.46	1.29–1.64	< 0.0001*
Non-ischaemic LGE	0.61	0.61	1.84	0.96–3.53	0.07
Number of segments of non-ischaemic LGE	0.14	0.086	1.15	0.97–1.36	0.11
LVEDVI	0.0076	0.0056	1.01	1.00-1.01	0.18
LVMI	0.019	0.0085	1.02	1.002–1.036	0.02*
LVEF	-0.016	0.014	0.98	0.96–1.01	0.24
RVEDVI	0.012	0.0089	0.01	0.99–1.03	0.17
RVEF	-0.012	0.013	0.99	0.96–1.01	0.36
LAVI	0.0086	0.0082	1.01	0.99–1.03	1.03
PTT	0.15	0.038	1.16	1.08–1.25	0.0001*
PBVI (scaled)	0.14	0.069	1.15	1.01–1.32	0.04*

*Signifies *p* < 0.05; BMI indicates body mass index, AF, atrial fibrillation; LGE, late gadolinium enhancement; LVEDV, left ventricular end diastolic volume; LVESV, left ventricular end systolic volume; LVEF, left ventricular ejection fraction; LVMI, left ventricular mass indexed; RVEDV, right ventricular end diastolic volume; RVESV, right ventricular end systolic volume; RVEF, right ventricular ejection fraction; LAVI, left atrial volume index; PTT, pulmonary transit time; PBVI, pulmonary blood volume indexed

Other factors that were associated with MACE on Cox regression analysis included age (HR 1.08, 95% CI 1.05–1.12, *P* < 0.001), NYHA class (HR 2.41, 95% CI 1.60–3.64, *P* < 0.001), diuretic use (HR 3.83, 95% CI 1.85–7.93, *P* = 0.0003), NT-proBNP level scaled (HR 1.46, 95% CI 1.29–1.64, *P* < 0.001, LV mass index (HR 1.02, 95% CI 1.002–1.036, *P* = 0.023) and PBVI (HR 1.001, 95%CI 1.001–1.002, *P* = 0.04) (Table 3).

On multivariate Cox regression, association between PTT and MACE remained significant after correction for age (HR 1.10, 95% CI 1.0083–1.189, *P* = 0.003), NYHA class (HR 1.14, 95% CI 1.06–1.50, *P* = 0.0006) and diuretic use (HR 1.15, 95% 1.06–1.24, *P* = 0.0005). However, the association was no longer significant when adjusted for all three factors (HR 1.08, 95% CI 0.99–1.17, *P* = 0.08) or NT-proBNP (HR 1.015, 95% CI 0.91–1.14, *P* = 0.79). PTT was independently associated with MACE even after correction for the only other prognostic CMR parameter, LVMI (Table 4).

**Table 4:** Multivariate cox-regression analysis of predictors of MACE and all-cause mortality

Covariate	Beta	SE	HR	95% CI of HR	*P* value
PTT	0.15	0.038	1.16	1.08–1.25	0.0001*
PTT (adjusted for age)	0.091	0.042	1.10	1.01–1.19	0.003*
PTT (adjusted for NYHA class)	0.13	0.038	1.14	1.06–1.50	0.0006*
PTT (adjusted for diuretic use)	0.14	0.039	1.15	1.06–1.24	0.0005*
PTT (adjusted for LVMI)	0.136	0.041	1.15	1.06–1.24	0.0009*
PTT (adjusted for NT-proBNP)	0.015	0.057	1.02	0.91–1.14	0.79

*Signifies *p* < 0.05; PTT indicates pulmonary transit time; NYHA, New York Heart Association; LGE, late gadolinium enhancement; LVMI, left ventricular mass indexed

## Discussion

### Pulmonary transit time and outcomes in heart failure

In this study we have shown that in patients with a recent diagnosis of heart failure, pulmonary transit time was associated with risk of MACE defined as all-cause death, heart failure admission, non-fatal stroke and non-fatal myocardial infarction. The majority of MACE events in patients with prolonged PTT were for heart failure hospitalisation or all-cause death. The association between PTT and MACE was independent of clinical risk factors such as age, NYHA class or diuretic use, and imaging risk factors including LVMI. There was a significant association between PTT and log transformed NT-proBNP and once adjusted for NT-proBNP level the association between PTT and MACE was no longer significant.

### Association between PTT and haemodynamic congestion

As the majority of heart failure hospitalisation is due to congestion, rather than low cardiac output, we propose that PTT may be used as a non-invasive haemodynamic marker of congestion and provide further evidence to support its prognostic utility in this group of patients [19]. In our study patients with prolonged PTT also had higher NYHA class, peripheral oedema and diuretic use suggesting a degree of clinical congestion. In addition, PTT was significantly associated with NT-proBNP, which is a marker of LV filling pressure and pulmonary capillary wedge pressure and is the most used test for quantification of congestion. The most accurate method for assessment of haemodynamic congestion is cardiac catheterisation by measurement of either pulmonary artery wedge pressure or left ventricular end-diastolic pressure. Previous studies have shown that PTT is correlated with these invasive haemodynamic indices and with haemodynamic congestion preceding clinical congestion, this may provide an avenue for therapeutic intervention to prevent heart failure hospitalisation [8, 18, 20].

### Optimal methodology for assessing PTT

PTT in our study was measured from arterial input function (AIF) images of dual sequence first-pass rest perfusion imaging without the requirement for additional imaging or contrast administration. Previous studies in heart failure have visually counted pulmonary transit time [5] whereas we have used a robust and validated artificial intelligence method [7, 14]. Conventional single sequence perfusion methods are optimised for signal intensity in the myocardium and risk signal saturation in the blood pool. In the dual sequence methodology, we have employed in this study, PTT was measured from the low-resolution AIF images where the signal is optimised for blood pool and there is less risk of signal saturation. This methodology is therefore potentially more accurate and easier to use in clinical practice.

In their study, Ricci et al. demonstrated that pulmonary blood volume (the product of stroke volume and pulmonary transit time in heart beats) was associated with severity of diastolic dysfunction as well as an increased risk of cardiovascular death, heart failure hospitalisation and significant ventricular arrhythmia. Houard et al. subsequently placed circular regions of interest in the RV and LV on first-pass perfusion imaging and generated time attenuation curves to measure the time difference between RV and LV bolus peaks [4]. Although this method demonstrated increased automation it still required manual input for placement of the regions of interest in the RV and LV blood pool. Using this method, the authors demonstrated that PTT had significant prognostic value in predicting overall mortality, cardiovascular death and heart failure hospitalisation in patients with advanced heart failure and reduced ejection fraction.

Following on from the above studies in patients with heart failure, our study has demonstrated the prognostic utility of PTT using an automated method that is robust and easy to use. Seraphim et al. demonstrated the prognostic value of this technique in an unselected cohort of patients referred for clinical myocardial perfusion assessment [7]. In contrast to our study, the median ejection fraction in this group of patients was 62% and they demonstrated that both PTT and PBVI independently predicted MACE.

### Clinical applications of PTT

Our study is the first to investigate the prognostic utility of a fully automated method for generating PTT by CMR in patients with heart failure. Investigation into the aetiology of LV dysfunction in patients with heart failure is one of the main indications for CMR with increasing use of stress perfusion testing in this group of patients [21]. If PTT can be obtained automatically during these studies, then it can provide additional prognostic information for “free”. We are not proposing that PTT replaces NT-proBNP but offers an additional assessment of congestion without any additional user input. In patients in whom stress perfusion imaging is not possible (e.g. asthma, haemodynamic instability) but are undergoing CMR for other purposes it may be possible to do rest perfusion imaging solely for the purpose of PTT quantification. In our study we found that as PTT increases, the risk of MACE and heart failure hospitalisation increases (Fig. 3). Patients with prolonged PTT may therefore warrant closer follow-up or medicine optimisation.

It is worth noting that current CMR does not incorporate any dynamic haemodynamic parameter. We speculate that volumetric assessment or tissue characterisation by CMR are less dynamic in nature when compared to PTT. Hence, PTT may offer a complimentary clinical role during routine multi-parametric CMR assessment. Currently echocardiography is the only non-invasive imaging modality to provide haemodynamic assessment of LV filling pressure [22]. PTT has been shown to be associated with diastolic function in patients with hypertrophic cardiomyopathy [17]. It may therefore have an important role to play in the assessment of patients with heart failure with reduced ejection fraction and those with preserved ejection fraction.

### Future studies

Future studies should be considered to further validate our results in independent heart failure populations. It will be important to define the normal range of PTT in healthy patients without heart failure. In our study there was significant overlap in PTT between patients with and without a MACE event and defining how this relates to normal ranges requires further work. There should also be a focus on interstudy reproducibility of measurement of PTT and subsequently whether PTT and the adverse outcomes associated with it can be altered by diuretics or other heart failure therapy. As PTT is influenced by left ventricular filling pressure, there may also be a role for its use in the diagnosis and risk stratification of patients with heart failure with preserved ejection fraction.

### Study limitations

This was a single centre study that focused on patients with presumed non-ischaemic cardiomyopathy. Patients with clinical or CMR evidence of ischaemic heart disease were not enrolled in this study, and so the results may not be applicable to those with LV dysfunction related to ischaemic heart disease. Prior to enrolment in this study, patients were not necessarily on optimal heart failure therapy, although there was no significant difference in the use of guideline directed medical therapy between those who did and did not suffer MACE. Our study did not have any invasive assessment of cardiac output or left ventricular end diastolic pressure, parameters which we know correlate with PTT.

During this study 4.5% of patients were excluded from analysis due to suboptimal LV fit during automated PTT assessment. This could be a limiting factor in the clinical integration of the technique which is otherwise automated. However, more recently changes have been made to increase the quality of the AIF and early data suggest the exclusion rate can be reduced to 2.5% or even further with ongoing improvements. This may help improve its ability to be rolled out for widespread use.

## Conclusions

PTT measured automatically during CMR perfusion imaging in patients with recent onset heart failure is an independent predictor of MACE and is strongly associated with heart failure hospitalisation and death. PTT derived in this way may be a non-invasive marker of haemodynamic congestion in heart failure. Future studies are required to establish if prolonged PTT identifies those who might benefit from increased diuretic therapy or other heart failure therapy.

## Data Availability

No datasets were generated or analysed during the current study.

## Abbreviations

AIF: Arterial input function
BSA: Body surface area
CMR: Cardiovascular magnetic resonance
ECV: Extracellular volume
HbA1c: Haemoglobin A1c
LA: Left atrium
LGE: Late gadolinium enhancement
LVEDV: Left ventricular end diastolic volume
LVEF: Left ventricular ejection fraction
MACE: Major adverse cardiovascular event
MOLLI: Modified look-locker inversion recovery
NYHA: New York Heart Association
PBV: Pulmonary blood volume
PTT: Pulmonary transit time
ROC: Receiver operating characteristic
RVEF: Right ventricular ejection fraction
